# Ribosome-free Terminals of Rough ER Allow Formation of STIM1 Puncta and Segregation of STIM1 from IP_3_ Receptors

**DOI:** 10.1016/j.cub.2009.07.072

**Published:** 2009-10-13

**Authors:** Gyorgy Lur, Lee P. Haynes, Ian A. Prior, Oleg V. Gerasimenko, Stefan Feske, Ole H. Petersen, Robert D. Burgoyne, Alexei V. Tepikin

**Affiliations:** 1 Department of Physiology, School of Biomedical Sciences, University of Liverpool, Liverpool L69 3BX, UK; 2 NYU Langone Medical Center, New York University, 522 First Avenue, New York, NY 10016, USA

**Keywords:** CELLBIO, SIGNALING

## Abstract

Store-operated Ca^2+^ entry is a ubiquitous mechanism that prevents the depletion of endoplasmic reticulum (ER) calcium [1]. A reduction of ER calcium triggers translocation of STIM proteins, which serve as calcium sensors in the ER, to subplasmalemmal puncta where they interact with and activate Orai channels ([2–8]; reviewed in [9]). In pancreatic acinar cells, inositol 1,4,5-trisphosphate (IP_3_) receptors populate the apical part of the ER. Here, however, we observe that STIM1 translocates exclusively to the lateral and basal regions following ER Ca^2+^ loss. This finding is paradoxical because the basal and lateral regions of the acinar cells contain rough ER (RER); the size of the ribosomes that decorate RER is larger than the distance that can be spanned by a STIM-Orai complex [5, 10], and STIM1 function should therefore not be possible. We resolve this paradox and characterize ribosome-free terminals of the RER that form junctions between the reticulum and the plasma membrane in the basal and lateral regions of the acinar cells. Our findings indicate that different ER compartments specialize in different calcium-handling functions (Ca^2+^ release and Ca^2+^ reloading) and that any potential interference between Ca^2+^ release and Ca^2+^ influx is minimized by the spatial separation of the two processes.

## Results and Discussion

### STIM1 Translocation in Live Pancreatic Acinar Cells

**Figure 1:**
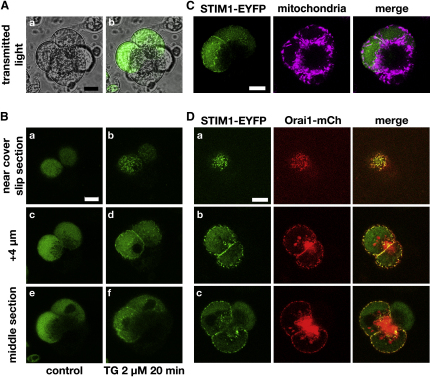
STIM1-EYFP Forms Basal and Lateral Puncta upon Ca^2+^ Store Depletion in Live Pancreatic Acinar Cells (Aa and Ab) A cluster of pancreatic acinar cells infected with STIM1-EYFP adenovirus. (Aa) Transmitted light image. Scale bars throughout Figure 1 represent 10 μm. (Ab) Overlay with EYFP fluorescence. (Ba–Bf) The same group of acinar cells as in (A), shown in three different confocal planes: nearest section to the coverslip (Ba and Bb), 4 μm from the coverslip toward the middle of the cells (Bc and Bd), and the middle section (approximately 10 μm from the coverslip) (Be and Bf). The columns show images of the cells before (left column) and after (right column) store depletion with 2 μM thapsigargin (TG). (C) Positioning of STIM1-EYFP puncta (green) with respect to mitochondria (magenta). The images show a middle confocal section of an acinar cell triplet following TG application. (Further details are shown in Figure S2.) (Da–Dc) Positioning of STIM1-EYFP puncta (green) with respect to Orai1-mCherry (red) in cells treated with 2 μM TG. Note copositioning of STIM1 puncta and Orai1 on lateral and basal segments of the plasma membrane (yellow). (Da) Nearest section to the coverslip. (Db) 4 μm from the coverslip toward the middle of the cells. (Dc) The middle section (approximately 10 μm from the coverslip).

We used primary isolated pancreatic acinar cells to study the localization and redistribution of the endoplasmic reticulum (ER) calcium sensor STIM1. We utilized an adenoviral construct of STIM1-YFP to transfect pancreatic acinar cells and observed translocation of STIM1 following depletion of ER calcium with a specific inhibitor of the sarcoplasmic/endoplasmic reticulum calcium pumps, thapsigargin (TG) (Figure 1). STIM1 formed subplasmalemmal puncta in both basal and lateral regions (Figure 1B; n = 11). The middle section of the cells (Figure 1Bf; see also Figure S1 available online) revealed a surprising absence of STIM1 puncta in the apical region. Pancreatic acinar cells contain a large number of secretory granules concentrated in the apical part of the cell, whereas the basal part is enriched with highly developed rough ER (RER) [11]. In this cell type, a perigranular mitochondrial belt is found on the boundary between the basal and apical regions [12]. STIM1 in ER-depleted cells did not form puncta in a region located apically with respect to the lateral mitochondria of the perigranular mitochondrial belt (Figure 1C; n = 11). Upon ER calcium depletion, we sometimes observed the formation of internal STIM1 puncta on the basal side of the mitochondrial belt (Figure 1C). This phenomenon was not further investigated.

Subplasmalemmal mitochondria are strategically positioned to buffer Ca^2+^ entering the cell via plasma membrane channels [13, 14]. There is a substantial body of evidence indicating the involvement of mitochondria in the regulation of store-operated Ca^2+^ entry (SOCE) ([15, 16]; reviewed in [1]). In our experiments, subplasmalemmal STIM1 puncta were frequently found in the vicinity of basal and lateral mitochondria (Figure 1C; Figure S2). STIM1 puncta and mitochondria did not inhabit the apical (secretory) part of the cell (Figure 1C). Orai1-mCherry expressed in the acinar cells was present in the apical region and on the lateral and basal plasma membrane (Figure 1D; Figure S3; see also legend of Figure S3). A similar distribution was found with antibodies against Orai1 (Figure S4). However, in TG-treated cells, we observed copositioning of STIM1 puncta and Orai1 in the basal and lateral but not the apical region of the plasma membrane (Figure 1D, particularly Figure 1Dc; n = 39), suggesting that SOCE mechanisms are assembled only in the basal and lateral plasma membrane regions.

### Calcium Store Depletion Triggers STIM1 Translocation Away from IP_3_ Receptors

**Figure 2:**
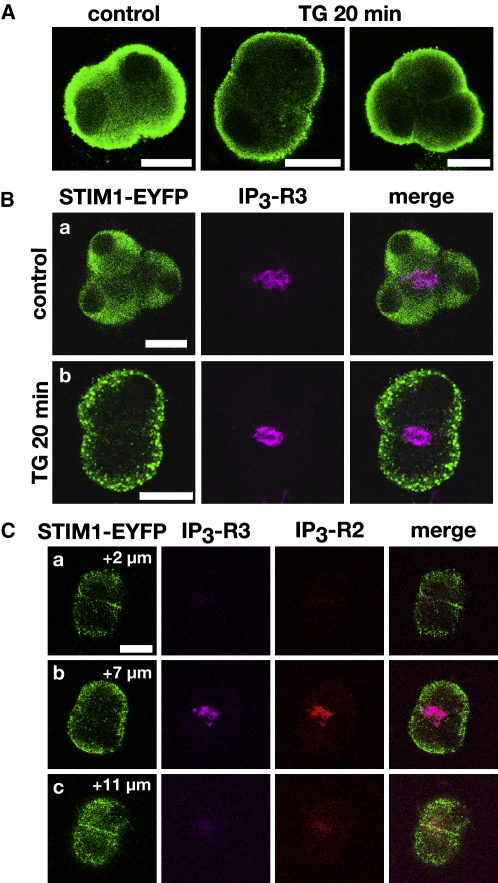
Positioning of STIM1 with Respect to IP_3_ Receptors in Fixed Pancreatic Acinar Cells (A) Immunostaining of acinar cells with antibodies against STIM1. Left panel shows the distribution of STIM1 in control (untreated) cells. Central and right images depict the distribution of STIM1 in cells treated with TG (in doublet and triplet, respectively). Scale bars throughout Figure 2 represent 10 μm. (Ba and Bb) Cells transfected with STIM1-EYFP were stained with anti-GFP (green) and anti-IP_3_ receptor subtype 3 (IP_3_-R3, magenta) antibodies. (Ba) Distribution of fluorescence in control conditions. (Bb) Distribution of fluorescence following TG treatment. (Ca–Cc) Confocal sections of an acinar cell doublet with the focal plane set below the granular region (Ca); in the middle of the cells, sectioning through the granular region (Cb); and above the granular region (Cc). STIM1-EYFP-transfected cells were fixed after TG-induced calcium store depletion and costained with anti-GFP (green), anti-IP_3_-R3 (magenta), and anti-IP_3_-R2 (red) antibodies. STIM1 translocated to the basolateral plasma membrane with apparent staining of the septum (lateral membrane) between the two cells in confocal sections under (Ca) or above (Cc) the granules. However, the middle section (Cb) shows that there are no STIM1 puncta in the apical region of the cells where the IP_3_ receptors are located.

We next probed the changes in the endogenous STIM1 distribution in fixed cells with STIM1 antibodies (gift from A. Rao, Harvard Medical School). As with live STIM-EYFP-expressing cells, in untransfected cells probed with STIM1 antibodies, we observed changes in localization of the fluorescent marker from a diffuse distribution (but excluded from nuclei) in untreated cells to a clear peripheral distribution in cells treated with TG (Figure 2A; n = 25). These experiments indicate that, following Ca^2+^ store depletion, STIM1 redistributes from the bulk ER to basal and lateral (but not apical) subplasmalemmal regions.

We next visualized the positioning of STIM1 with respect to inositol 1,4,5-trisphosphate (IP_3_) receptors. The IP_3_ receptors in pancreatic acinar cells are localized in the apical region of the cell [17, 18]. Immunostaining cells before and after TG treatment revealed changes in the positioning of STIM1 in relation to IP_3_ receptors. The IP_3_ receptors responsible for Ca^2+^ signaling and Ca^2+^-dependent secretion in pancreatic acinar cells are type 2 (IP_3_-R2) and type 3 (IP_3_-R3) [19]. Both types of receptors were observed clearly in the apical part of the cell (Figures 2B and 2C; Figure S5A). In the resting cells (untreated by TG), STIM1 could be observed in all parts of the cell except the nuclei (Figure 2Ba). The apical staining of STIM1 was usually weaker than in basal regions, indicating a lower density of ER in the apical region (well documented for this cell type [20]). Some ER strands were present in the apical region, and at-rest STIM1 staining was observed in the region containing IP_3_-Rs (Figure 2Ba). Following TG treatment, the difference in localization of IP_3_-Rs and STIM1 became more pronounced (Figure 2Cb; n = 14), with IP_3_-R3 (Figure 2Bb; n = 45) and IP_3_-R2 (Figure S5Aii; n = 17) still localized in the apical part of the cell while STIM1 moved away from the IP_3_-Rs to the basal and lateral membranes. Strikingly, no colocalization was observed between STIM1 and either type of IP_3_ receptor following TG treatment (Figures 2B and 2C; Figure S5Aii).

A direct involvement of IP_3_ receptors in the regulation of SOCE has previously been suggested in an elegant conformational-coupling hypothesis [21, 22]. However, the molecule responsible for sensing the ER Ca^2+^ concentration is not the IP_3_ receptor but STIM. The pancreatic acinar cells provide an extreme example of disconnection between the IP_3_ receptors and the activator of store-operated calcium influx STIM1 (Figure 2).

**Figure 3:**
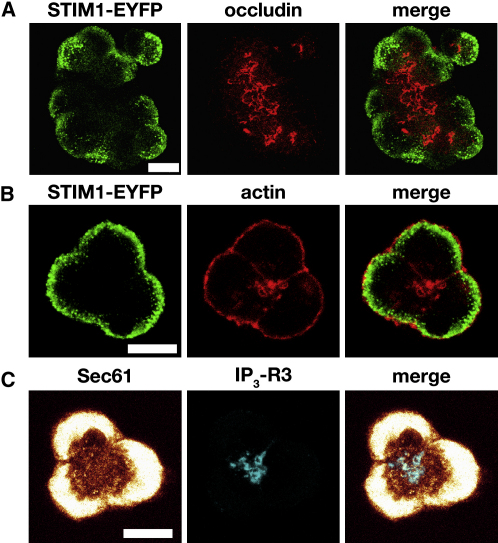
Relative Positioning of STIM1, Tight Junctions, and Actin Filaments (A) STIM1 and occludin. STIM1-EYFP-transfected acinar cells were fixed after TG treatment and stained with anti-GFP (green) and anti-occludin (red) antibodies. The image shows the middle confocal section through a larger cluster of acinar cells. Scale bars throughout Figure 3 represent 10 μm. (B) STIM1-EYFP-transfected acinar cells were fixed after TG treatment and stained with anti-GFP (green) and anti-β-actin (red) antibodies. (C) Relative positioning of ER strands (revealed by antibodies against Sec61, yellow) and IP_3_-R3 (blue).

In pancreatic acinar cells, tight junctions segregate the apical portion of the plasma membrane from the lateral and basal parts [23]. The apparent escape of STIM1 from the apical compartment (Figure 2) prompted us to investigate the relative positioning of STIM1 and the tight junctions. We used antibodies against occludin, an essential junction protein [24], to label tight junctions in STIM1-EYFP-expressing cells (Figure 3A; n = 64). Following ER calcium store depletion, STIM1 aggregated basally with respect to the tight junction (occludin staining), and colocalization of the two proteins was not observed (Figure 3A; Figure S5B).

An important hallmark of the apical region is a high density of filamentous actin (F-actin), which partially colocalizes with the IP_3_-Rs [17]. Figure 3B illustrates that following TG treatment, STIM1 does not colocalize with the apical F-actin (n = 12).

It is important to note that although STIM1 does not form puncta in the apical region, strands of ER are present in this cellular compartment and partially colocalize with IP_3_-Rs (Figure 3C; n = 12). These IP_3_-R- containing ER strands are located very close to the apical plasma membrane but are somehow prevented from making subplasmalemmal STIM1 puncta.

In live pancreatic acinar cells, we observed and characterized STIM1 translocation induced not only by TG but also by the calcium-releasing secretagogue cholecystokinin (CCK) (10 pM), the neurotransmitter acetylcholine (ACh) (50 nM), and the inhibitors of energy production oligomycin (5 μM) and iodoacetate (2 mM) as well as the bile acid taurolithocholic acid 3-sulphate (TLC-S) (Figure S6). Importantly, for all tested physiological and pathological stimuli, subplasmalemmal STIM1 puncta were formed in basal and lateral regions. Similarly to the experiments with TG (see Figure 1, Figure 2, and Figure 3), we did not observe puncta formation in the apical region (data not shown). It is interesting to note that low concentrations of CCK and ACh induce local cytosolic Ca^2+^ signals in the apical part of the cell [25, 26]—the region occupied by IP_3_ receptors ([17, 18]; reviewed in [20])—yet these very secretagogues trigger translocation of STIM1 to basal and lateral regions of the cell.

### Rough ER Makes Plasma Membrane Junctions by Removing Ribosomes from the Contact Sites

**Figure 4:**
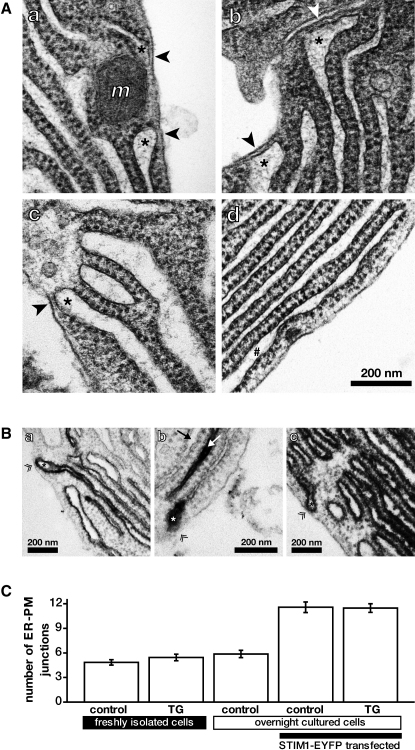
Electron Microscopy of Ribosome-free ER Terminals and ER-Plasma Membrane Junctions (Aa–Ad) Ribosome-free ER terminals and ER-plasma membrane (ER-PM) junctions are indicated by black or white arrowheads (depending on the background). ER strands forming the ribosome-free terminals in (Aa)–(Ac) are indicated by asterisks (^∗^). Mitochondria are indicated by *m*. Scale bar in (Ad) is applicable to (Aa)–(Ad). All images are from basal or lateral regions. (Aa) Ribosome-free terminals and ER-PM junctions in freshly isolated acinar cells. (Ab) ER-PM junctions in STIM1-EYFP-transfected pancreatic acinar cells with intact ER calcium store. (Ac) ER-PM junctions in STIM1-EYFP-transfected cells following TG treatment. (Ad) The parts of the basal and lateral subplasmalemmal regions where the terminals are absent. In this panel, the rough ER lumen closest to the plasma membrane is indicated by a #. (Ba–Bc) Dark precipitates in ER lumen are formed in cells infected by HRP-STIM1-containing adenovirus. The strands of ER approaching the plasma membrane are indicated by asterisks (^∗^); junctions are identified by double arrowheads (≫). All images are from basal or lateral regions of TG-treated cells. (Ba) Some, but not all, ER strands are filled with a dark precipitate, and gradients of staining can be observed. (Bb) Example of ER strand approaching the PM with most of the staining concentrated in the region adjacent to the PM. White arrow indicates ER lumen completely filled with DAB precipitate; black arrow indicates ER lumen that is apparently clear of precipitate. (Bc) Example showing all ER strands filled with DAB precipitate. (C) Number of ER-PM junctions per electron microscopy cell section (± standard error of the mean). The cell treatments are outlined below the graph; images of cells following some of these treatments are shown in (A). Note the increase in junction numbers following transfection with STIM1-EYFP.

The delegation of the calcium reloading function to the basolateral part of the acinar cell is potentially problematic. In pancreatic acinar cells, this cellular region is populated by RER ([11]; Figure 4A). Ribosomes that define this ER compartment are large structures, 26 ± 3 nm (n = 80) in pancreatic acinar cells. This would be enough to prevent formation of STIM1-Orai1 complexes and preclude activation of SOCE. Figure 4 shows how the acinar cells bypass this problem. We observed that on approach to the plasma membrane, strands of RER lose ribosomes and form ribosome-free terminals adjacent to the plasma membrane region (indicated by arrowheads in Figure 4A). The minimum distance between the ER membrane and the plasma membrane in these junctions is only 12–13 nm—substantially smaller than the size of a ribosome (see Table S1). This distance is in good agreement with both the estimate by Várnai and colleagues (11–14 nm) based on introducing linkers between the plasma membrane and the ER [10] and the estimate of approximately 17 nm by Wu and colleagues [27]. The appearance of these ribosome-free terminals was similar in freshly isolated control cells (untreated with TG and untransfected with STIM1-EYFP; see Figure 4Aa), cells treated with TG for 20 min before fixation (data not shown), cells transfected with STIM1-EYFP but untreated with TG (Figure 4Ab), cells transfected with STIM1-EYFP and treated with TG (Figure 4Ac; for enlarged fragment showing a ribosome-free RER terminal, see Figure S7A), and untreated cells incubated overnight in conditions used for STIM1-EYFP transfection but without virus (data not shown). The minimum distance between the ER membrane in the ribosome-free terminals and the plasma membrane was also similar under all of these conditions (Table S1). Although the appearance of ribosome-free terminals and the minimum distance between the plasma membrane and the ER membrane in the junctions were similar in control cells and in cells transfected with STIM1-EYFP, the density of the terminals was different: cells expressing STIM-EYFP had an approximately 2-fold higher density of terminals (Figure 4C). This suggests that STIM1 could be involved in formation of ribosome-free terminals and ER-plasma membrane (ER-PM) junctions. Treatment with TG did not appear to produce any difference in the density of the terminals (Figure 4C). In agreement with the light microscopy results, ribosome-free terminals were found in basal and lateral regions of the cell, but not in the region enriched with secretory granules (data not shown). In those parts of the basal and lateral subplasmalemmal regions where the ribosome-free terminals were absent (see Figure 4Ad), the minimum distance between the membrane of the RER and the plasma membrane was more than 47 nm. The minimum distance in these terminal-free regions was similar for all conditions described above (Table S2). This distance was larger than the estimated size of a ribosome and larger than the previously estimated intermembrane distance spanned by STIM-Orai complexes [10, 27]. The ribosome-free terminals (shown in Figures 4Aa–4Ac) were the only observed sites where the distance between the ER and the plasma membrane was sufficiently small to provide the platforms for STIM1 interaction with the plasma membrane structures.

Transfection of cells with adenoviruses encoding HRP-STIM1 (gift from R. Lewis, Stanford University [27]) allowed us to test for the presence of STIM1 in the ER lumen, and particularly in ER terminals approaching the plasma membrane (Figure 4B; n = 24). Depending on the efficiency of transfection, we observed the formation of precipitates in some ER strands (including ER strands approaching the plasma membrane, Figures 4Ba and 4Bb; for enlarged fragment of Figure 4Ba showing a ribosome-free RER terminal, see Figure S7B) or in all ER strands (Figure 4Bc). For lower levels of transfection, it was possible to observe gradients of staining in different parts of the ER (Figures 4Ba and 4Bb). Dark precipitates were observed in both TG-treated cells (Figure 4B) and control cells (untreated with TG; see Figure S8 and legend of Figure S8). The precipitates in the ER lumen were observed only in cells infected with HRP-STIM1 adenovirus. The results of these experiments suggest that STIM1 indeed populates ER-PM junctions.

### Conclusions

Achieving coordination between Ca^2+^ release from the ER and Ca^2+^ reloading, the cells have developed ribosome-free extensions of RER, which allow this organelle to form close junctions with the basolateral plasma membrane so that the ER Ca^2+^ content can be “reported” by STIM1 to the plasma membrane effectors. The ribosomes are removed specifically from the area of the junction but decorate the rest of the RER. This mechanism allows formation of ER-PM junctions in the regions dominated by RER, i.e., in basal and lateral regions distant from the IP_3_-Rs in the apical region.

We have previously shown that the ER in pancreatic acinar cells is lumenally continuous [28]. The present study demonstrates why IP_3_-Rs cannot be directly involved in SOCE and that the lumenally continuous ER forms separate domains specializing in different elements of calcium homeostasis—calcium release and calcium reloading. Both Ca^2+^ release and Ca^2+^ influx are regulated by cytosolic Ca^2+^; therefore, spatial segregation between IP_3_-Rs and STIM1-activated channels could be a mechanism for reducing interference between Ca^2+^ release and Ca^2+^ influx. To our knowledge, this is the first time such segregation has been reported for any type of polarized secretory cell.

By removing the SOCE mechanism from the apical membrane, secreting cells improve conditions for vectorial transport of Ca^2+^ (i.e., Ca^2+^ extruded from the cell will not be reabsorbed via these channels). This observation could have general significance for other secretory epithelia. Surprisingly, the RER, which is responsible for protein synthesis, folding, and transport, is also employed in activating Ca^2+^ influx. The pancreatic acinar cell has evolved an economical design maximizing the surface area of its protein-producing organelle by employing the same organelle for Ca^2+^ reloading. It is likely that this design is followed in other types of exocrine secretory cells and more generally in cell types specializing in a high level of protein synthesis.

## Experimental Procedures

### Cell Isolation and Transfection

Pancreatic acinar cells were isolated from pancreata of adult CD-1 mice (25–35 g) as described previously [29]. Cells were then seeded on 35 mm glass-bottom culture dishes and cultured in modified Na-HEPES-based standard extracellular solution containing (in mM) NaCl (140), KCl (4.7), HEPES (10), MgCl_2_ (1), glucose (10), CaCl_2_ (1) (pH 7.4) supplemented with MEM amino acids, 292 μg/ml L-glutamine, 100 units/ml penicillin, 100 μg/ml streptomycin, and trypsin inhibitor (3 mg/ml) (pH 7.4). Cells derived from one pancreas were seeded equally onto six dishes and infected with a virus concentration of 5.4 × 10^6^ pfu/ml per dish and incubated overnight (12–14 hr) at 35°C.

### Plasmid Constructs and Viruses

The STIM1-EYFP and Orai1-mCherry constructs were produced as described previously [30]. A construct encoding an HRP-tagged version of STIM1 in which the HRP tag was inserted immediately following the STIM1 signal sequence [27] was used as a template to amplify the HRP-STIM1 cassette by polymerase chain reaction with primers containing restriction endonuclease sites to permit subsequent subcloning into pcDNA3.1(+) expression vectors. STIM1-EYFP, HRP-STIM1-pcDNA, and Orai1-mCherry adenovirus constructs were produced by Vector Biolabs (Philadelphia).

### Confocal Microscopy of Live Cells

To ensure minimal STIM1 overexpression, experiments were conducted not more than 12–14 hr after infection. For live-cell imaging, glass-bottom dishes were mounted in a perfusion chamber on the stage of a Leica TCS SL inverted confocal microscope equipped with a 63× oil immersion objective (NA 1.3). Cell culture medium was changed to Na-HEPES-based standard extracellular solution. TG, inhibitors of ATP production, and Ca^2+^-releasing agonists were added via gravity-fed perfusion system at the specified concentrations. To visualize mitochondria, cells were loaded with tetramethylrhodamine methyl ester. For further information on excitation/emission and loading protocols, see Supplemental Experimental Procedures.

### Immunostaining

To visualize the distribution of endogenous STIM1, we used noncommercial STIM1 antibodies [31] in 1:100 dilution on acinar cells fixed in 4% paraformaldehyde (PFA). To investigate the relative positioning of IP_3_ receptors and STIM1, we transfected cells with STIM1-EYFP and fixed them in ice-cold methanol. STIM1 localization was revealed with primary antibodies against GFP (1:200, Invitrogen). IP_3_-Rs and tight junctions were stained with antibodies against IP_3_-R3 (BD Transduction Laboratories) and/or IP_3_-R2 (Chemicon International, Millipore) or occludin (Zymed Laboratories, Invitrogen) at a concentration of 1:100, 1:50, and 1:100, respectively. Actin was labeled with a monoclonal anti-β-actin antibody (Sigma) and rough ER was visualized with Sec61 antibodies (Cambridge Bioscience), both used at 1:100 dilution. Appropriate secondary antibodies conjugated to Alexa 488 and/or Alexa 594 and 647 (Invitrogen) were used at a dilution of 1:1000 and 1:500, respectively. (Spectral properties and further details of procedures can be found in Supplemental Experimental Procedures.)

### Electron Microscopy

For conventional electron microscopy, cells were fixed for 1 hr in 4% PFA and 2.5% glutaraldehyde at room temperature and then postfixed with 1% osmium tetroxide and stained with 0.5% uranyl acetate. Following this, cells were flat-embedded in Agar 100 epoxy resin. Ultrathin 60–80 nm sections were cut and transferred onto formvar-coated 200-mesh copper grids. Samples were then counterstained with 4% uranyl acetate and Reynold's lead citrate before visualizing on a 100 kV Tecnai G2 Spirit electron microscope. Images were taken at 43,000×–87,000× magnification.

HRP-STIM1-transfected acinar cells were seeded on to grid-lined glass-bottom culture dishes (MatTek) and fixed in a gradient of 2%–4% PFA at 35°C. HRP signal was amplified with a TSA-biotin system (PerkinElmer) and ABC kit (Vector Laboratories). After amplification, cells were incubated with DAB peroxidase substrate (Vector Laboratories) for 30 min in Tris-buffered saline. Cells forming precipitates were located on the grid via light microscopy and processed for flat embedding. Serial ultrathin (60 nm) sections of the identified cells were transferred to 100-mesh copper grids and visualized without counterstaining.

## Supplemental Data

Document S1. Supplemental Experimental Procedures, Two Tables, and Eight Figures
