# Supplementary material for: Ribosome-free Terminals of Rough ER Allow Formation of STIM1 Puncta and Segregation of STIM1 from IP3 Receptors
Source: Curr Biol. 2009 Oct 13;19(19):1648–53. doi: 10.1016/j.cub.2009.07.072 (PMC2887489; doi:10.1016/j.cub.2009.07.072)
Supplement: Document S1. Supplemental Experimental Procedures, Two Tables, and Eight Figures [file mmc1.pdf]

Current Biology, Volume *19*

Supplemental Data

Ribosome-free Terminals of Rough ER Allow Formation of STIM1 Puncta and Segregation of STIM1 from IP_3 Receptors

Gyorgy Lur, Lee P. Haynes, Ian A. Prior, Oleg V. Gerasimenko, Stefan Feske, Ole H. Petersen, Robert D. Burgoyne, and Alexei V. Tepikin

Supplemental Experimental Procedures

Plasmid Constructs and Viruses

STIM1-EYFP, HRP-STIM1-pcDNA and Orai1-mCherry adenovirus constructs (in replication deficient adenoviruses) were produced by Vector Biolabs(Philadelphia, PA, USA). Briefly, the entire coding region from each vector was sub-cloned into the pDual-CCM shuttle vector (Vector Biolabs) for transfer into the adenovirus genome vector. Recombinant adenovirus particles were amplified using standard protocols and titrated to determine the concentration of active adenoviruses present.

Confocal Microscopy of Live Cells

Images of STIM1-EYFP were obtained with a 514nm excitation light and emitted light was collected using 520-550nm bandpass filter. Orai-mCherry fluorescence was excited at 594nm and emitted light was collected with a 600-650nm filter. To visualize mitochondria, live cells were loaded with 25nM TMRM for 5 minutes and imaged using a 543nm excitation light. Emission light in these experiments was collected using 560-600nm bandpass filter. The plasma membrane of the cells (see Figure S1) was visualized using FM 4-64 dye excited at 514nm and emission was collected with a 675-775nm filter.

Immunostaining

For endogenous STIM1 staining cells were fixed with 4% paraformaldehyde for 30 minutes. After permeabilisation with 0.1% Triton X-100 nonspecific antibody binding was blocked with 10% goat serum and 1% BSA for 60 minutes at room temperature. Cells were stained with anti-STIM1 primary antibody (1:100) for 2 hours and anti-rabbit Alexa488 conjugated secondary antibody for 30 minutes at room temperature. Cover slips were mounted using ProlongGold (Invitrogen, Paisley, UK) mounting medium.

We and others [1] found that methanol fixation was the best protocol for immunostaining of IP_3 receptors. However using this fixation technique we have not been able to visualise the translocation of endogenous STIM1 with either commercial or non-commercial STIM1 antibodies. To investigate the relative positioning of IP_3 receptors and STIM1 we had to use cells transfected with STIM1-EYFP and antibodies against EYFP together with IP_3-R antibodies.

Cells were fixed in 100% cold methanol at -20°C for 10 minutes. Following blocking procedure (see above) cells were incubated with primary antibodies against the proteins mentioned in the main text. Orai 1 was stained with a non-commercial antibody obtained from Dr. Stefan Feske used in 1:100 dilution for 1 hour at room temperature. After secondary antibody staining for 20 minutes at room temperature cover slips were mounted on microscope slides with ProlongGold (Invitrogen, Paisley, UK). Confocal sections were spaced at 0.5μm in axial directions throughout the whole cell (from near cover slip section to the top of the cell). Alexa 488 dye was excited at 488nm and emitted light was collected at 500-540nm, Alexa 594 was excited at 543nm and a 580-620nm bandpass filter was used whilst Alexa 647 was excited at 633nm and emission was collected with a 640-700nm bandpass filter.

Sources of Common Chemicals and Reagents

Tris-buffered saline was from BioRad (Hercules, CA, USA). Cell culture reagents, trypsin inhibitor, TMRM, FM 4-64 were purchased from Invitrogen (Paisley, UK). Collagenase was from Worthington (Lorne Laboratories, Reading, UK). Thapsigargin was from Calbiochem (San Diego, CA, USA). Paraformaldehyde, glutaraldehyde, osmium tetroxide, uranyl acetate, lead citrate, epoxy resin, formvar and 100 and 200 mesh copper grids was from Agar Scientific (Stansted, UK). All other chemicals were from SIGMA (Gillingham, UK).

Table S1. ER-PM Distances in Junctions

	Mean Minimum Distance (nm)	SEM	n Total
Freshly isolated, control	13.0	0.6	13
Freshly isolated, treated with TG	13.5	1.0	8
Overnight cultured, control	12.3	0.6	20
Overnight cultured, STIM1-EYFP transfected, control	12.2	0.8	8
Overnight cultured, STIM1-EYFP transfected, treated with TG	12.1	0.5	22

Tables S1 and S2 represent measurements of minimum distances between ER and PM. A value for the minimum distance was measured for every junction. The data were then averaged.

Table S2. ER-PM Distances in Nonjunction Areas

	Mean Minimum Distance (nm)	SEM	n Total
Freshly isolated, control	53.3	2.2	34
Freshly isolated, treated with TG	48.3	3.3	18
Overnight cultured, control	49.0	2.0	26
Overnight cultured, STIM1-EYFP transfected, control	47.8	4.8	9
Overnight cultured, STIM1-EYFP transfected, treated with TG	50.5	2.4	30

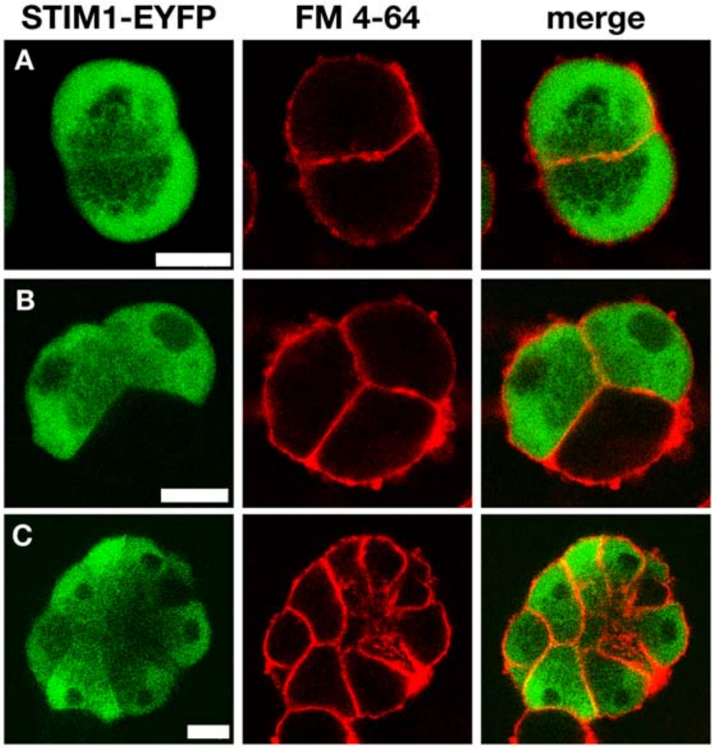

Figure S1. Distribution of STIM1-EYFP and FM 4-64 in Live Unstimulated Pancreatic Acinar Cells

Under control conditions (i.e. cells not treated with TG or Ca^{2+}-releasing agonists) STIM1-EYFP (green) fluorescence is not concentrated in puncta, instead it demonstrates a typical ER distribution, well-documented for this cell type, [2-5]. The fluorescence is higher in the basal region of the cells where an extensive network of the ER is located and is lower in the apical region where only some strands of ER penetrate into the granular (apical) region. The secretory granule region of the pancreatic acinar cell was reported to produce strong light scattering[6,7]. To image plasma membrane structures in this region we stained the cells with the lipophilic dye FM 4-64 (shown in red). Images show the middle confocal section of an acinar cell (A) doublet, a (B) triplet and a (C) larger cluster. All components of the plasma membrane (including the apical region) are clearly visible. Scale bars correspond to 10μm.

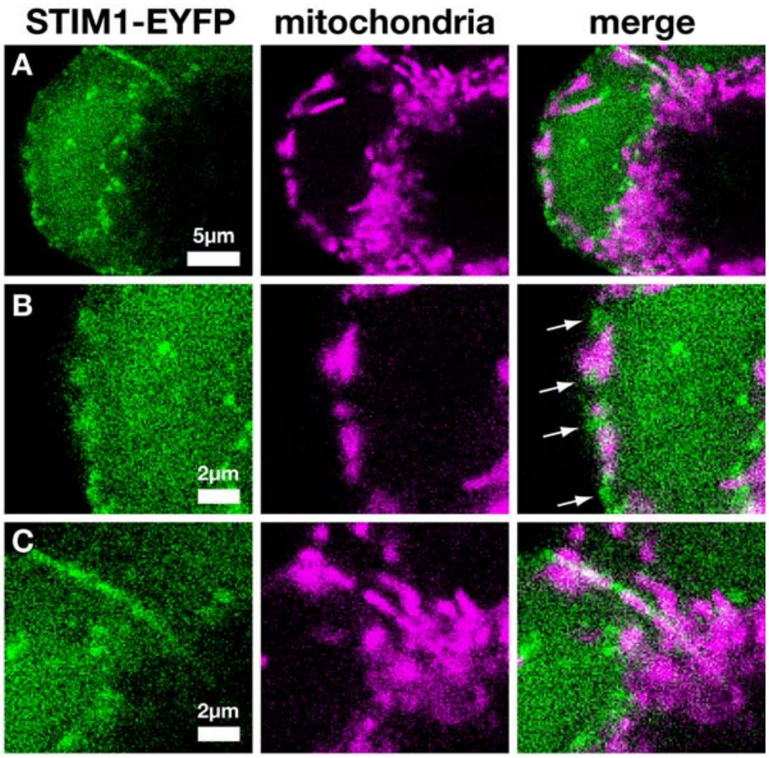

Figure S2. Relative Positioning of STIM1 and Mitochondria in Pancreatic Acinar Cells treated with TG

(A) Enlarged fragments of image shown in Figure 1C. The fragment shows one of the STIM1 –expressing cells from the cluster. Scale bar is 5μm.

(B) An enlarged fragment of the image (A) showing the basal membrane where STIM1 puncta appear to be in close proximity to subplasmalemmal mitochondria (arrows pointing at STIM1 puncta on the merged image). Scale bar is 2μm.

(C) Enlarged fragment of the image (A) showing STIM1-EYFP translocated to the lateral membrane. Sub-plasmalemmal STIM1 puncta appear to be ‘embedded’ into the subplasmalemmal mitochondria. No STIM1 puncta are seen apically with respect to the perigranular mitochondria. Scale bar corresponds to 2μm.

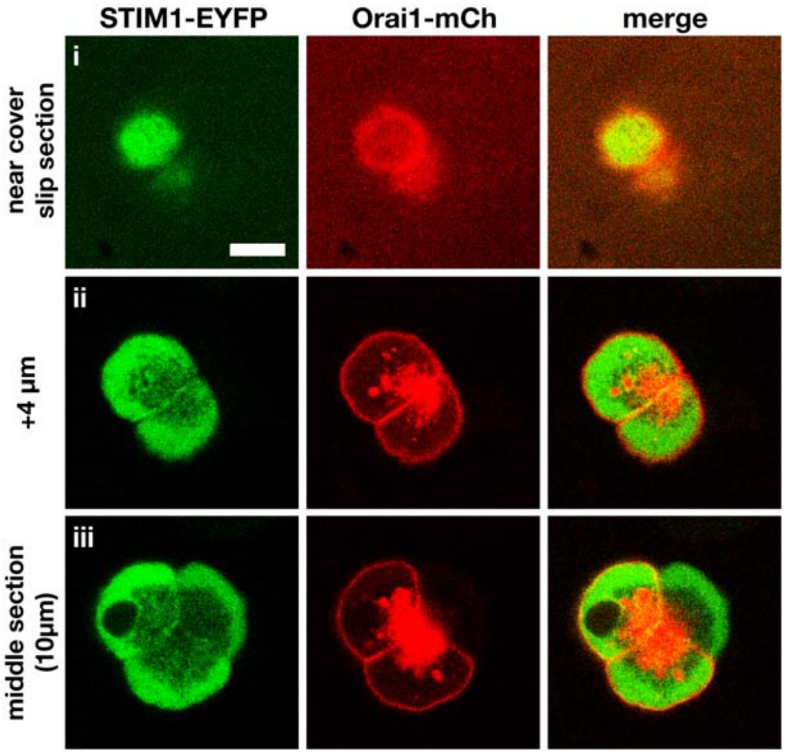

Figure S3. Positioning of STIM1-EYFP (Green) with Respect to Orai1-mCh (Red) in Cells with Intact Ca^{2+} Stores (Untreated with TG)

Scale bar corresponds to 10 µm. This figure shows the same cluster of pancreatic acinar cells as Figure 1D. (i) Shows confocal section nearest to the cover slip, (ii) 4µm from the cover slip towards the middle of the cells and (iii) the middle section (approximately 10µm from the cover slip). Note diffuse distribution of STIM1-EYFP and uniform staining of basolateral membrane with Orai1-mCh. Note the changes of distribution of both constructs from uniform to punctuate following the TG treatment (compare with Figure 1D). The apical region contains high level of Orai1-mCh (ii and iii of this figure). This region also contains IP_3-Rs (Figure 2) and we can not exclude that the two proteins could co-localise and / or interact in this region. We consider that the relationship of Orai1 and IP_3-Rs in pancreatic acinar cells should be addressed in a separate study. The important conclusion from the experiments illustrated by this figure and by Figure 1D is that STIM1 and Orai1 transfected into acinar cells using adenoviral constructs change distribution upon depletion of Ca^{2+} store and that co-clustering of STIM1 and Orai1 is observed in the basal and lateral, but not apical regions of the plasma membrane.

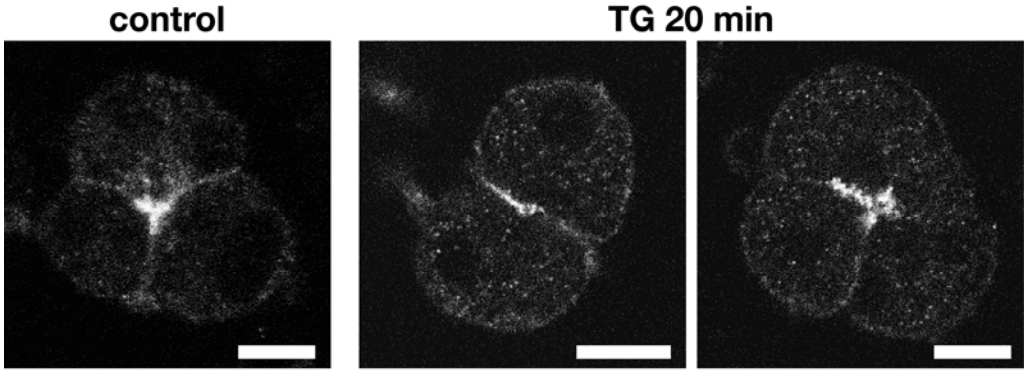

Figure S4. Distribution of Orai1 in Pancreatic Acinar Cells

The images were obtained by immunostaining of pancreatic acinar cells with polyclonal antibodies against the C terminus (aa278-294) of Orai1 (developed by S.F.). Scale bar corresponds to 10μm. The left panel shows the distribution of Orai1 in control cells (not treated with TG; n = 34 cells), whilst central and right panels show the distribution in cells treated with TG (2μM, n = 27 cells). Note the high level of fluorescence in the apical region and fainter staining of lateral and basal regions of the plasma membrane. The distribution of endogenous Orai1 revealed using antibodies in permeabilized cells (this figure) was similar to that found using Orai1-mCh expressing live cells (see Figure 1D). The immunostaining was however too faint to investigate the clustering of endogenous Orai1.

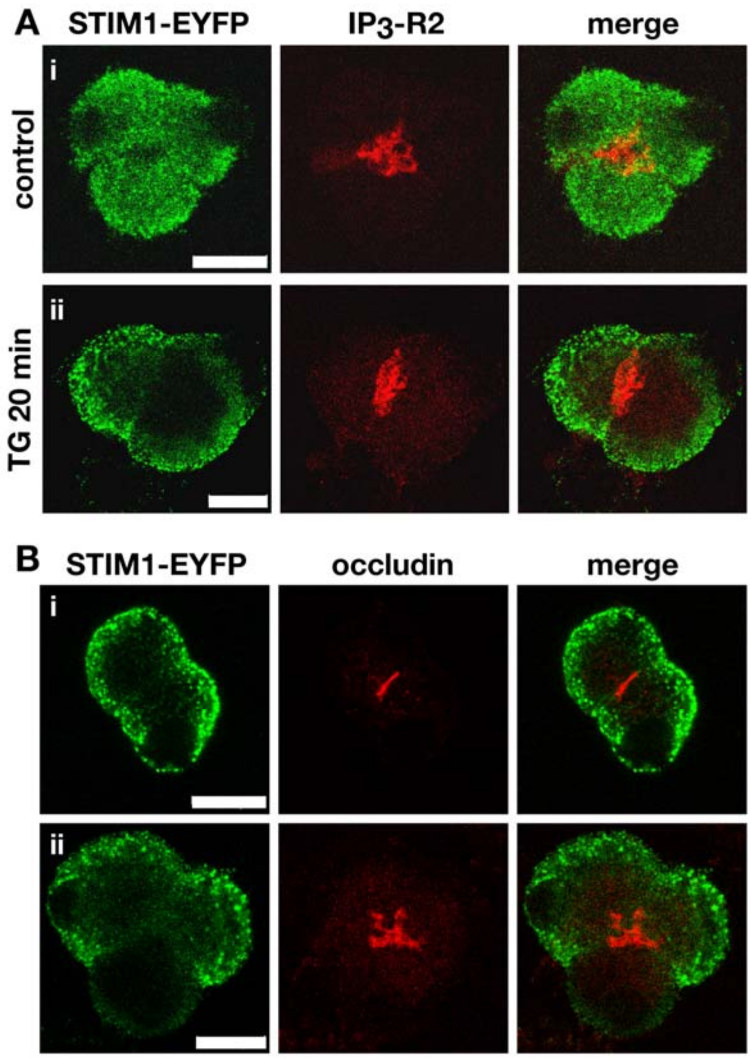

Figure S5. Relative Positioning of STIM1, IP_3-R2 and Tight Junctions in Doublets and Triplets of Acinar Cells

Scale bars correspond to 10µm.

(A) Cells stained with anti-GFP (green) and anti-IP_3-receptor subtype 2 (red) antibodies under (i) control and (ii) store depleted conditions. Note apical localisation of IP_3-R2 and basolateral localization of STIM1 in TG-treated cells.

(B) STIM1-EYFP transfected acinar cells were fixed after thapsigargin treatment and stained with anti-GFP (green) and anti-occludin (red)

The images show the middle confocal section through (i) a doublet and (ii) a triplet of pancreatic acinar cells. The distribution of STIM1 and occludin in large clusters is illustrated in Figure 3A.

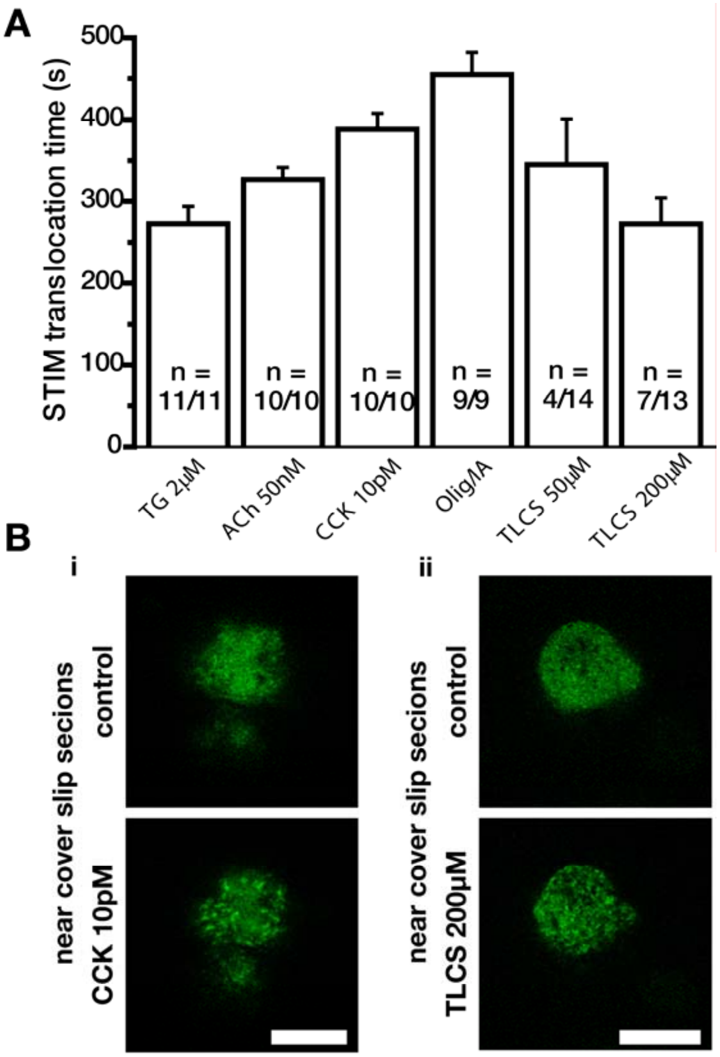

Figure S6. STIM1-EYFP Translocation into Puncta upon Store Depletion with Various Agents

Formation of STIM1 puncta in pancreatic acinar cells was observed not only due to depletion of ER calcium with TG but also following application of a low concentration of ACh, a low (physiological) concentration of CCK, inhibition of energy production with Oligomycin (5µM) and Iodoacetate (2mM) as well as following treatment with the bile acid taurolithocholic acid 3-sulfate (TLC-S) which is considered to be a putative trigger for an important disease, acute pancreatitis. Experiments summarised in this figure were conducted on live pancreatic acinar cells in the presence of 1mM Ca^{2+} in the extracellular solution.

(A) The histogram shows the time necessary for the formation of the first STIM1 puncta for individual stimuli (together with the corresponding standard error). The first number on the individual bars indicates the number of experiments in which the formation of puncta was observed, the second number indicates the total number of experiments with a particular Ca^{2+} releaser. Only 4 out of 14 cells showed puncta formation following application of 50µM TLC-S and 7 out of 13 cells for 200µM TLC-S. All cells formed STIM1 puncta when treated with ACh or CCK or Olig/IA.

(B) The optical section nearest to the cover slip was used for the early detection of STIM1 puncta formation. The scale bars correspond to 10µm. The upper images show the distribution of STIM1-EYFP fluorescence in resting cells (near-cover slip optical section). The lower images illustrate formation of puncta induced by CCK or TLC-S in the same optical section.

Notably, the first STIM1 puncta induced by low concentrations of secretagogues developed 300-400s after the addition of CCK or ACh. This is a relatively short time with respect to the periods of physiological stimulation of pancreatic secretion. This and the low doses of physiological agonists that were effective suggest the involvement of store-operated calcium channels in stimulus-secretion coupling in pancreatic acinar cells.

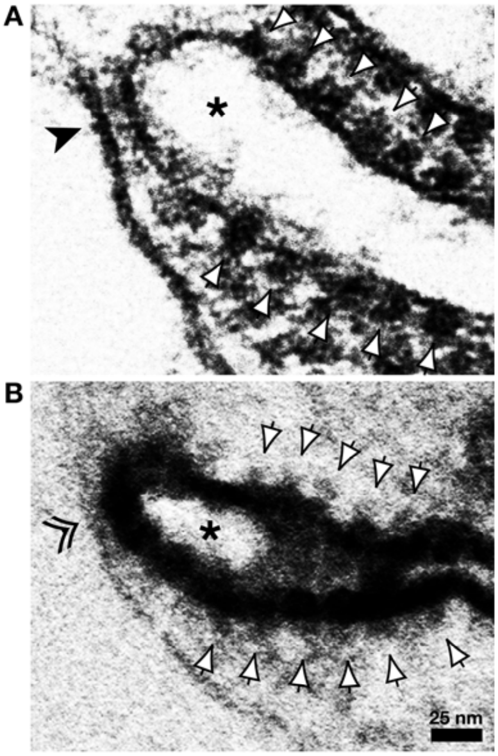

Figure S7. Electron Microscopy of Ribosome-free ER Terminals Forming ER-PM Junctions

Enlarged fragments of images depicted on Figure 4. ER strands approaching the plasma membrane are indicated by symbol *. Ribosomes, decorating the ER strands outside the junctional regions, are indicated by white arrows.

(A) This is an expanded fragment from Figure 4Ac. Arrowhead indicates the ER-PM junction. The scale bar is applicable to both images.

(B) This is an expanded fragment from Figure 4Ba. The darkened lumen of the ER is the result of HRP-STIM1 expression. Note the disappearance of ribosomes in the vicinity of the junctions.

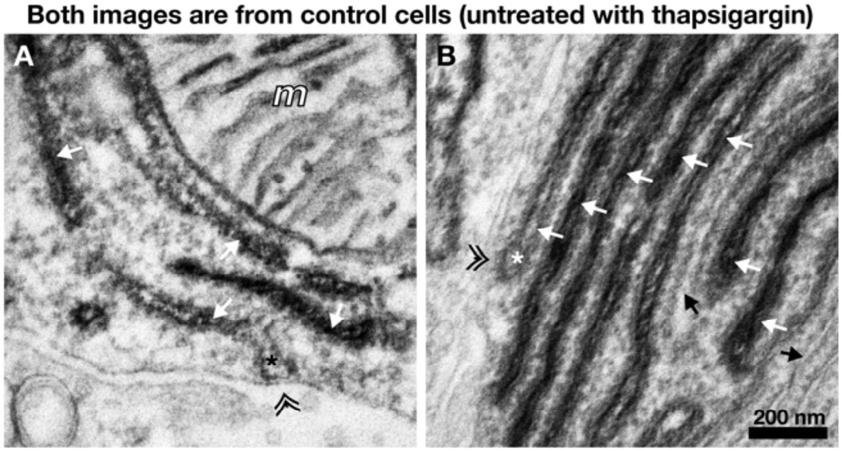

Figure S8. Electron Microscopy of ER Strands and ER-PM Junctions in Control (Untreated with TG) Cells

White arrows show ER strands decorated with DAB precipitate, black arrows identify ER strands which are apparently clear from precipitate. Mitochondria are indicated by: ***m***. The strands of ER approaching the plasma membrane are highlighted by symbol *****. The ER-PM junctions are identified by double arrowheads. Scale bar (in B) is applicable to both panels of the figure. Note that these control cells (not treated with TG, n = 14) show HRP-dependent precipitates in the ER strands (similarly to TG treated cells, Figure 4). This is in line with the study of Wu and colleagues from Richard Lewis laboratory [8].

Supplemental References

1. Turvey M.R., Fogarty K.E., and Thorn P. (2005). Inositol (1,4,5)-trisphosphate receptor links to filamentous actin are important for generating local Ca2+ signals in pancreatic acinar cells**.** J. Cell Sci. *118***:** 971-980.
2. Bolender R.P. (1974). Stereological analysis of the guinea pig pancreas. I. Analytical model and quantitative description of nonstimulated pancreatic exocrine cells**.** J. Cell Biol. *61***:** 269-287.
3. Park M.K., Petersen O.H., and Tepikin A.V. (2000). The endoplasmic reticulum as one continuous Ca(2+) pool: visualization of rapid Ca(2+) movements and equilibration**.** EMBO J. *19***:** 5729-5739.
4. Gerasimenko O.V., Gerasimenko J.V., Rizzuto R.R., Treiman M., Tepikin A.V., and Petersen O.H. (2002). The distribution of the endoplasmic reticulum in living pancreatic acinar cells**.** Cell Calcium *32***:** 261-268.
5. Dolman N.J., Gerasimenko J.V., Gerasimenko O.V., Voronina S.G., Petersen O.H., and Tepikin A.V. (2005). Stable Golgi-mitochondria complexes and formation of Golgi Ca(2+) gradients in pancreatic acinar cells**.** J. Biol. Chem. *280***:** 15794-15799.
6. Nemoto T., Kojima T., Oshima A., Bito H., and Kasai H. (2004). Stabilization of exocytosis by dynamic F-actin coating of zymogen granules in pancreatic acini**.** J. Biol. Chem. *279***:** 37544-37550.
7. Sherwood M.W., Prior I.A., Voronina S.G., Barrow S.L., Woodsmith J.D., Gerasimenko O.V., Petersen O.H., and Tepikin A.V. (2007). Activation of trypsinogen in large endocytic vacuoles of pancreatic acinar cells**.** Proc. Natl. Acad. Sci. U. S. A *104***:** 5674-5679.
8. Wu M.M., Buchanan J., Luik R.M., and Lewis R.S. (2006). Ca2+ store depletion causes STIM1 to accumulate in ER regions closely associated with the plasma membrane**.** J. Cell Biol. *174***:** 803-813.
